# A Deep Learning Framework to Predict Tumor Tissue-of-Origin Based on Copy Number Alteration

**DOI:** 10.3389/fbioe.2020.00701

**Published:** 2020-08-05

**Authors:** Ying Liang, Haifeng Wang, Jialiang Yang, Xiong Li, Chan Dai, Peng Shao, Geng Tian, Bo Wang, Yinglong Wang

**Affiliations:** ^1^College of Computer and Information Engineering, Jiangxi Agricultural University, Nanchang, China; ^2^Department of Urology, Shanghai East Hospital, Tongji University School of Medicine, Shanghai, China; ^3^Geneis (Beijing) Co. Ltd., Beijing, China; ^4^School of Software, East China Jiaotong University, Nanchang, China

**Keywords:** tumor, tissue-of-origin, copy number alteration, autoencoder, convolution neural network

## Abstract

Cancer of unknown primary site (CUPS) is a type of metastatic tumor for which the sites of tumor origin cannot be determined. Precise diagnosis of the tissue origin for metastatic CUPS is crucial for developing treatment schemes to improve patient prognosis. Recently, there have been many studies using various cancer biomarkers to predict the tissue-of-origin (TOO) of CUPS. However, only a very few of them use copy number alteration (CNA) to trance TOO. In this paper, a two-step computational framework called CNA_origin is introduced to predict the tissue-of-origin of a tumor from its gene CNA levels. CNA_origin set up an intellectual deep-learning network mainly composed of an autoencoder and a convolution neural network (CNN). Based on real datasets released from the public database, CNA_origin had an overall accuracy of 83.81% on 10-fold cross-validation and 79% on independent datasets for predicting tumor origin, which improved the accuracy by 7.75 and 9.72% compared with the method published in a previous paper. Our results suggested that the autoencoder model can extract key characteristics of CNA and that the CNN classifier model developed in this study can predict the origin of tumors robustly and effectively. CNA_origin was written in Python and can be downloaded from https://github.com/YingLianghnu/CNA_origin.

## 1. Introduction

Cancer metastasis is the process in which tumor cells fall off from the primary site, enter the circulatory system, transfer to other parts of the body, and continue to grow. In about 3–5% of metastatic tumors, the sites of origin cannot be found, and this is known as cancer of unknown primary site (CUPS). Patients diagnosed with CUPS are treated with broad-spectrum anticancer drugs and have a low median survival time of 9–12 months. Precise diagnosis of the tissue of origin for metastatic CUP is essential for deciding on the treatment scheme to improve the patient's prognosis (Chen et al., 2017). Clinical, imaging and pathological examination are used to detect the tissue of origin, but these approaches can only determine the tissue of origin in about 50–80% of CUP patients.

Recently, a large number of studies have tried to use cancer biomarkers to predict the primary tumor site for CUPs so as to provide much-needed guidelines for timely patient care and cancer therapy (Liang et al., 2016; Grewal et al., 2019; Wang et al., 2019; Zheng et al., 2019). The gene expression patterns in tumors have high specificity, and so these the most widely used biomarkers for tumor classification (Bloom et al., 2004; Tothill et al., 2005; Staub et al., 2010; Wu et al., 2010; Handorf et al., 2013; Xu et al., 2016; Wang et al., 2018; Li et al., 2019). For example, Li used the within-sample relative gene expression orderings of gene pairs within individual samples to identify a prediction signature (Li et al., 2019). Wang proposed a general framework to identify a subset of genes for each tumor subtype and presented a corresponding classification model for distinguishing different tumor subtypes (Wang et al., 2018). Xu established a comprehensive database integrating microarray- and sequencing-based gene expression profiles of 16,674 tumor samples covering 22 common human tumor types to discriminate the origins of tumor tissue, which will be an additional useful tool for determining the tumor origin (Xu et al., 2016).

DNA methylation and miRNA regulate the expression of genes involved in numerous biological processes (Rosenfeld et al., 2008; Rosenwald et al., 2010; Ferracin et al., 2011; Mueller et al., 2011; Søkilde et al., 2014). Tang developed a user-friendly webserver to predict tumor origin by identifying highly tissue-specific CpG sites and miRNA expression (Tang et al., 2017). Bae tried to discover tissue-specific methylation markers and predicted the tissue-of-origin in CUPS (Bae et al., 2018). Yang proposed an inverse space sparse representation model to distinguish tumor origins considering the characteristics of gene-based tumor data (Yang et al., 2019). Visual imagery is one of the main methods used by pathologists to assess the stage, type, and subtype of tumors (Shi et al., 2016; Coudray et al., 2018; Mohsen et al., 2018). Coudray employed visual inspection of histopathology slides to classify lung adenocarcinoma, lung squamous cell carcinoma, and normal lung tissue, which achieved performance comparable to that of pathologists (Coudray et al., 2018). Ultrasound imaging can also be used for tumor detection and diagnosis with a deep polynomial network algorithm (Shi et al., 2016).

As yet, few studies have investigated the roles of genome variants on tissue-of-origin in CUPS. Genome variants include mutation, small insertion, and deletion (INEDL) and copy number alteration (CNA). CNA is amplification and deletion of genomic sequences ranging from kilobases (Kb) to megabases (Mb) in size, which covers 360 Mb and encompasses hundreds of genes, disease loci, and functional elements (Redon et al., 2006). As the main genetic marker of the genome, CNA can affect the gene function through gene dose, gene breakage, gene fusion, and position effects and is closely related to the occurrence and development of tumor (Poduri et al., 2013). CNA also plays an increasingly important role in targeted therapy, personalized treatment, and prognosis judgment for tumors. Marquard developed a tool named TumorTracer by using publicly available somatic mutation data to train random forest classifiers and thus to identify the tissue of origin. This was demonstrated to be accurate enough to aid in the clinical diagnosis of cancers with unknown primary origin (Marquard et al., 2015). Zhang conducted a comprehensive genome-wide analysis of CNAs from six cancer types and selected 19 discriminative genes for tumor classification, but their overall prediction accuracy was about 75% (Zhang et al., 2016). In the current study, a computational method called CNA_origin is proposed to predict the tissue of origin with the information of gene CNA levels. CNA_origin set up an intellectual deep-learning network mainly composed of an autoencoder and a convolution neural network (CNN). This predictor successfully learned the inherent information of gene copy number and exhibited superior performance to classical algorithms for the same benchmark datasets.

## 2. Materials and Methods

### 2.1. Datasets

The copy number signal was produced by Affymetrix SNP 6.0 arrays for the set of samples in the cancer genome atlas (TCGA) study, as generated with the Firehose analysis pipeline. The preprocessing analysis of the dataset was performed with GISTIC (Beroukhim et al., 2007). These datasets were from primary solid tumor samples released by MSKCC in 2013 that could be downloaded from http://cbio.mskcc.org/cancergenomics/pancan_tcga/. The datasets with a sample size greater than 400 were selected. The details of all tissue samples, including tumor status, histopathology details, and sample sizes, are summarized in Table 1.

**Table 1:** Number of samples per tissue for CNA profiles.

**Primary site**	**Histology**	**CNA datasets**
Breast	BRCA (Breast invasive carcinoma)	847
Colorectal	COADREAD (Colorectal adenocarcinoma)	575
Brain	GBM (Glioblastoma multiforme)	563
Kidney	KIRC (Kidney renal clear cell carcinoma)	490
Ovarian	OV (Ovarian serous cystadenocarcinoma)	562
Uterine	UCEC (Uterine Corpus Endometrial Carcinoma)	443

Each sample had 24,174 genes with discrete copy number values denoted as “–2,” “–1,” “0,” “1,” “2,” where “–2” was homozygous deletion, “–1” was heterozygous loss, “0” was diploid, “1” was one copy gain and “2” was high-level amplification or multiple-copy gain (Ciriello et al., 2013). The CNA values were scaled to [–1, 1] with Equation (1).

(1) $$\begin{array}{r} x^{\prime }=\frac{x}{|x{|}_{max}} \end{array}$$

where x was the CNA value of the gene, |*x*|_*max*_ was the maximum absolute value of CNA among samples, and *x*′ was the value after correction.

### 2.2. Feature Extraction

**Figure 1:**
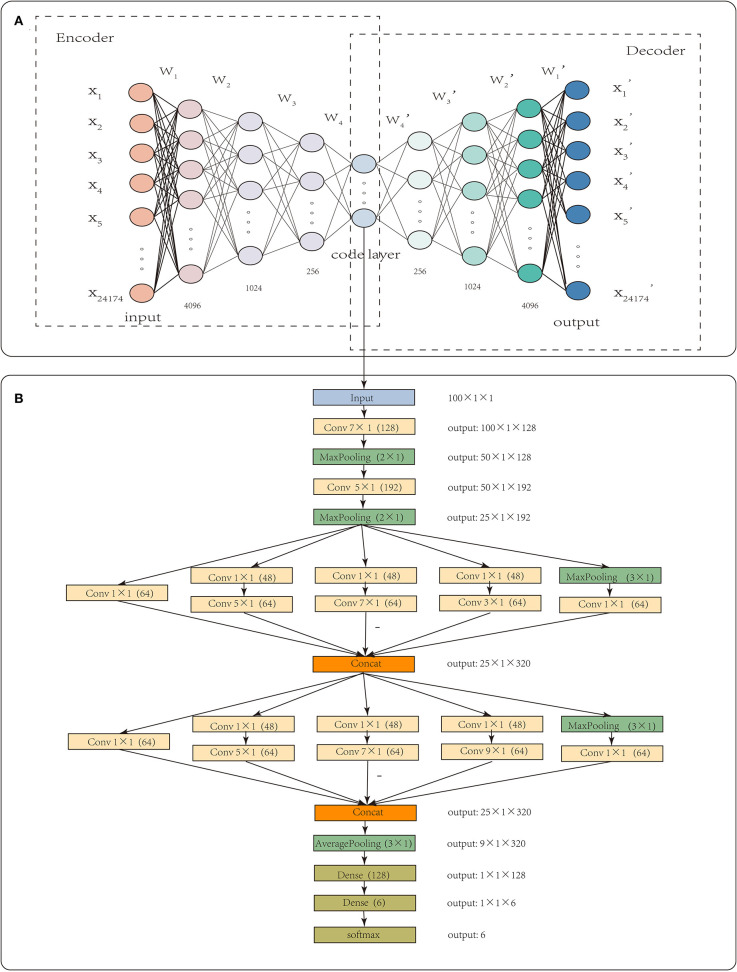
The workflow of CNA_origin. CNA_origin applied a stacked autoencoder to extract the feature of CNA values, which was composed of a symmetrical encoder and decoder network, and 4,096, 1,024, and 256 were the neuron numbers in symmetrical hidden layers **(A)**. A 1D CNN with multi-scale convolution kernels (1 × 1, 3 × 1, 5 × 1, 7 × 1, 9 × 1) was used to construct a classifier model, and the number 48 or 64 in parenthesis behind *k* × 1 meant convolution with 48 or 96 filters. The Concat layer stacked features from each branch together; the output denoted the dimensions of feature maps for each layer **(B)**.

Each sample had 24,174 gene-level CNA values. High dimensionality and small sample sizes have seriously obscured the intrinsic nature of CNA data. In this paper, CNA_origin applied a stacked autoencoder (SAE) to extract the features of CNA values, which converted the high-dimensional data into low-dimensional codes by training a multilayer neural network with small central layers to reconstruct high-dimensional input vectors (Hinton and Salakhutdinov, 2006). The SAE consisted of an adaptive multilayer “encoder” network and an asymmetric “decoder” network, and high-dimensional abstraction whilst maintaining the key information was achieved for feature reduction with the help of hidden nodes in the code layer, as illustrated in Figure 1A.

In the encoder network, the 24,174 gene-level CNA values used as inputs were mapped to the latent representation of next layer using Equation (2).

(2) $$\begin{array}{r} X^{\left[i\right]}=f\left(W_{i}X^{\left[i-1\right]}+b_{i}\right) \end{array}$$

where *f*(*x*) = *max*(0, *x*) was ReLU activation function, *b*_*i*_ was the bias of layer i, and *W*_*i*_ was the weight between layer i-1 and i. In the decoder network, the code layer was used to reconstruct the input by a reverse mapping using Equation (3).

(3) $$\begin{array}{r} X^{\left[i\right]}=f\left(W_{i}^{\prime }X^{\left[i-1\right]}+b_{i}^{\prime }\right) \end{array}$$

where $W_{i}^{\prime }$. The tanh activation function $f\left(x\right)=\frac{e^{x}-e^{-x}}{e^{x}+e^{-x}}$ was added to predict the final value, and the dimensionality of the final output layer was the same as that of the input layer. To determine the optimized parameters of W and b, layer-by-layer pretraining was used to minimize the error between the input X and output *X*′. The middle features were extracted through hidden nodes in the code layer.

CNA_origin was implemented in Python 3.7.3 using Keras (2.24) with the backend of TensorFlow (1.14.0). For the feature extraction of gene CNA, the neuron numbers in symmetrical hidden layers were set at 4,096, 1,024, 256, 100, 256, 1,024, and 4,096, respectively. The middlemost 100 neurons represented the extracted features, as it was found that features with more than 100 dimensions were not helpful to improve the classifier performance. The initial learning rate was set to 0.01, batch size to 64, and epochs to 16. This autoencoder was optimized using the Adam algorithm to learn the model parameters, and the loss function was mean square error.

### 2.3. Classifier Construction

The fully connected layer learns the global patterns in feature space, but convolution layer applies filters in the form of convolution operations to learn local patterns from the image (Baek et al., 2018). Inspired by the visual world, CNN has two interesting properties, translation invariant and spatial hierarchies of patterns, which allow a convolution network to efficiently learn increasingly complex and abstract visual concepts (Chollet, 2015, 2017). These properties are specialized for image data and also show outstanding performance in sequence processing (Le et al., 2017, 2019b). The same input transformation was performed on every subsequence; a pattern learned at a certain position in a sequence was later recognized at a different position, making 1D convnets translation invariant. A 1D convolution layer could catch local patterns in a sequence, making it competitive with recurrent neural networks (RNN) on sequence-processing at a considerably cheaper computational cost.

CNA_origin reshaped the 100 features of the sample into a 100 × 1 vector; each input tensor was 100 in width, 1 in height, and 1 in depth. The 1D convolution was used to extract local subsequences with D filters, and each filter was of *k* × 1 in size, which means the filter was k in width and 1 in height. CNA_origin utilized multi-scale convolution kernels, such as 1 × 1, 3 × 1, 5 × 1, 7 × 1, and 9 × 1, to extract high-order features of different levels and increase the diversity of feature extraction. Among them, the 1 × 1 convolution kernel changed the number of channels, increased the non-linear transformation of features, and improved the generalization ability of the network. The number 48 or 64 in parentheses behind *k* × 1 meant convolution with 48 or 96 filters. CNA_origin padded the features by adding k/2 columns with elements being zero to the head and tail of the sequence; therefore, the width of the new sequence after convolution with stride 1 was still the same.

The Concat operation in Figure 1 meant that the layer stacked features from each branch together. Different convolution layers and max-pooling layers concatenated like the Inception module, which increased the depth of the network and improved the robustness of the CNN. At the beginning of the network, a larger convolution kernel was used to reduce the number of parameters and computation, as illustrated in Figure 1B. In the last, the network connected two full connection layers, with a dropout layer to avoid overfitting. Usually, the number of hidden units was far larger than the obtained data, resulting in overfitting. The dropout layer helped alleviate this problem by removing some of the connections in the network (Baek et al., 2018). Output such as 50 × 1 × 128 meant that the feature maps were 50 in width, 1 in height, and 128 in depth. The final result was the probability that the sample belonged to each class and was found with the “softmax” activation function, which is often used in solving multi-classification problems. It was defined as Equation (4).

(4) $$\begin{array}{r} P_{k}=\frac{exp\left(\alpha_{k}\right)}{\sum_{i\;=\;1}^{m}exp\left(\alpha_{i}\right)} \end{array}$$

*P*_*k*_ was the probability that the sample belonged to class k. exp(x) represented an exponential function, α_*k*_ was the input value of class k, and m was the number of tumor classes. The categorical cross-entropy loss corresponding with the “softmax” activation function was used, which was a variant of binary cross-entropy and was defined as Equation (5).

(5) $$\begin{array}{r} loss=-\overset{n}{\underset{i\;=\;1}{\sum }}y_{i1}logP_{i1}+y_{i2}logP_{i2}+\cdots +y_{im}logP_{im} \end{array}$$

*P*_*im*_ was the predicted probability, n was the number of samples, and *y*_*im*_ was the true label.

For the classification learning, the number of multi-scale convolution kernels was set to 64, batch size to 16, and epochs to 12. The learning rate was dynamically adjusted according to the loss value of the test dataset, and the initial value was 0.01. The dropout rate was set to 0.4, and the loss function was sparse categorical crossentropy.

## 3. Results and Discussion

### 3.1. Performance Evaluation Metrics

The six tumor datasets were used to train CNA_origin. To understand the generalization performance, CNA_origin was also tested by independent datasets. In this work, the precision (P), recall (R), accuracy (ACC), and F1-score were adopted to assess the performance of the corresponding method; they have been used as measurement metrics in previous works (Le et al., 2018, 2019a). They are defined as Equation (6).

(6) $$\begin{array}{r} \;P=\frac{T_{P}}{T_{P}+F_{P}}\\ \;R=\frac{T_{P}}{T_{P}+F_{n}}\\ \;ACC=\frac{T_{P}+T_{n}}{T_{P}+F_{p}+F_{n}+T_{n}}\\ F1-score=\frac{2\times P\times R}{P+R} \end{array}$$

where *T*_*P*_, *T*_*n*_, *F*_*P*_, and *F*_*n*_ were the numbers of true positives, true negatives, false positives, and false negatives, respectively. *P* ∈ [0, 1], *R* ∈ [0, 1], *ACC* ∈ [0, 1], and *F*1−*score* ∈ [0, 1]. P = 0 indicated that all predicted positive results were actually negative. When all results were incorrect, *T*_*P*_ = 0 and *T*_*n*_ = 0; therefore, P = 0, R = 0, ACC = 0, and F1-score = 0. When all results were correct, *F*_*P*_ = 0 and *F*_*n*_ = 0; therefore, P = 1, R = 1, ACC = 1, and F1-score = 1. Precision and recall are two contradictory metrics. Generally speaking, when the precision is high, the recall is often low, while when the recall is high, the precision is often low.

### 3.2. CNA_Origin Performance

Ten-fold cross-validation was utilized to evaluate our algorithm with the extracted 100-dimensional features. The datasets were randomly divided into ten subsets of approximately equal size. Our network was trained 10 times; nine of the 10 subsets were used as the training datasets, and the remaining one was the test dataset. All of the above evaluation indices of our algorithm, that is, P, R, ACC, and F1-score, were calculated according to the results in our work. The average values of four metrics P, R, ACC, and F1-score defined in Equation (6) over ten test datasets are listed in Table 2.

**Table 2:** CNA_origin performance measured by three metrics via 10-fold cross-validation.

**Cancer**	**Precision**	**Recall**	**F1-score**
BRCA	0.8750	0.9231	0.8984
COADREAD	0.8158	0.7381	0.7750
GBM	0.9310	0.8438	0.8852
KIRC	0.8889	0.9600	0.9231
OV	0.8980	0.8672	0.8800
UCEC	0.6792	0.7200	0.6990

### 3.3. Performance Comparison With Other Algorithms

The performance of our algorithm was compared with four other classical classification algorithms with the same benchmark datasets. Random forest (RF) is an ensemble classifier that produces multiple decision trees using a randomly selected subset of training samples and variables (Liu et al., 2019). XGBoost is a novel sparsity-aware algorithm for sparse data and weighted quantile sketch for approximate tree learning and has been used in many bioinformatics fields (Chen and Guestrin, 2016; Deng et al., 2020; Hu et al., 2020). Long Short-Term Memory (LSTM) is an artificial RNN architecture that is well-suited to classifying, processing, and making predictions based on time series data (Hochreiter and Schmidhuber, 1997). Zhang proposed a method to computationally classify cancer types by using CNA level values; this was denoted as CNA_zhang here because the authors did not give the method a name (Zhang et al., 2016). CNA_zhang used minimum redundancy maximum relevance (mRMR) and incremental feature selection (IFS) to select features and the Dagging algorithm to give the final classification. The input of LSTM, RF, and XGboost was the extracted features from the autoencoder, and the GridSearchCV function in the sklearn package was used to select the optimal super-parameters that, were promised in the best condition.

Table 3 shows that the performance of CNA_origin was superior to LSTM, RF, XGboost, and CNA_zhang for BRCA, KIRC, OV, and UCEC. For BRCA, compared with LSTM and CNA_zhang, the F1-score was increased by 4.6 and 8.1%, respectively, and the recall (R) was increased by 9.08 and 5.67%, respectively. For GBM, CNA_origin performed slightly worse than the best, XGboost, with reductions of 2.35% in precision, 5.32% in recall, and 3.91% in F1-score. For KIRC, compared with LSTM and CNA_zhang, the F1-score was increased by 2.02 and 6.88%, respectively, and the recall was increased by 3.58%. For UCEC, compared with LSTM and CNA_zhang, the F1-score was increased by 3.97 and 21.56%, respectively, and the recall was increased by 9.80 and 53.41%, respectively. For COADREAD, CNA_origin performed slightly worse than the best LSTM algorithm, with reductions of 4.81% in precision, 8.61% in recall, and 6.81% in F1-score, respectively. For OV, the F1-score of CNA_origin was increased by 4.50% and 10.00% compared with LSTM and CNA_zhang; the recall was worse than the best, LSTM, by 5.10%, and precision was better than LSTM and CNA_zhang by 14.49 and 6.13%, respectively. CNA_origin exhibited perfect performance for the tumor classification.

**Table 3:** Comparison of CNA_origin predictions with those of other algorithms.

**Cancer**	**Predictor**	**Precision**	**Recall**	**F1-score**
BRCA	CNA_origin	**0.8750**	**0.9231**	**0.8984**
	LSTM	0.8713	0.8462	0.8585
	RF	0.8556	0.8645	0.8601
	XGboost	0.8214	0.8846	0.8519
	CNA_zhang	0.7916	0.8735	0.8306
COADREAD	CNA_origin	0.8158	0.7381	0.7750
	LSTM	**0.8571**	**0.8077**	**0.8317**
	RF	0.7659	0.6923	0.7272
	XGboost	0.7959	0.7500	0.7723
	CNA_zhang	0.6000	0.7346	0.6605
GBM	CNA_origin	0.9310	0.8438	0.8852
	LSTM	0.8913	0.8913	0.8913
	RF	0.8627	0.8627	0.8627
	XGboost	**0.9535**	**0.8913**	**0.9213**
	CNA_zhang	0.8870	0.8593	0.8730
KIRC	CNA_origin	0.8889	**0.9600**	**0.9231**
	LSTM	0.8837	0.9268	0.9048
	RF	**0.9056**	0.8571	0.8807
	XGboost	0.8780	0.8780	0.8780
	CNA_zhang	0.8085	0.9268	0.8636
OV	CNA_origin	**0.8980**	0.8627	**0.8800**
	LSTM	0.7843	**0.9091**	0.8421
	RF	0.7826	0.9000	0.8372
	XGboost	0.7551	0.8409	0.7957
	CNA_zhang	0.8461	0.7586	0.8000
UCEC	CNA_origin	0.6792	**0.7200**	**0.6990**
	LSTM	0.6897	0.6557	0.6723
	RF	0.6451	0.6060	0.6250
	XGboost	0.7407	0.6557	0.6957
	CNA_zhang	**0.7419**	0.4693	0.5750

*The bold values are the best performance among counterparts*.

**Figure 2:**
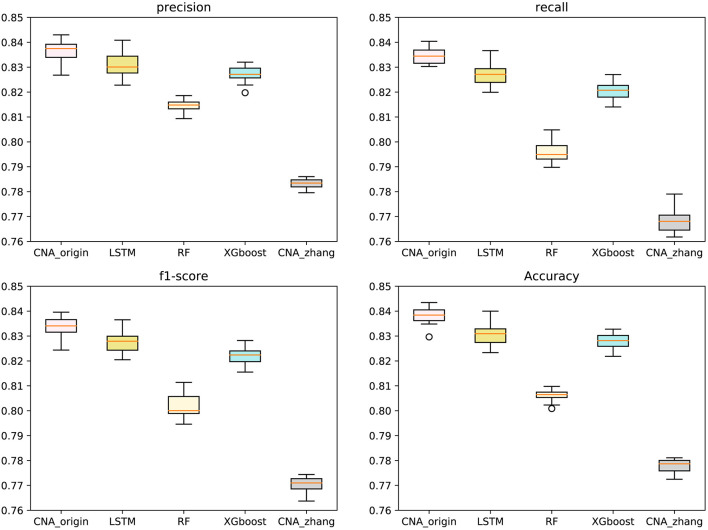
Performance comparison between CNA_origin and other algorithms (basic LSTM, RF, XGboost, and CNA_zhang) for the macro-averages of precision, F1-score, recall, and accuracy from 10-fold cross-validation 100 times.

The macro-averages of precision, F1-score, recall, and accuracy of six types of tumors were utilized to evaluate our predictor. Ten-fold cross-validation was run 100 times to test CNA_origin, LSTM, RF, XGboost, and CNA_zhang. For precision, CNA_origin had a mean value of 0.8369, which was increased by 0.70 and 6.87% compared with LSTM and CNA_zhang. For recall, the mean value of CNA_origin was 0.8345, which was increased by 0.91 and 8.68% compared with LSTM and CNA_zhang, respectively. For the F1-score, the mean value of CNA_origin was 0.8339, which was increased by 0.77 and 8.22% compared with LSTM and CNA_zhang, respectively. For accuracy, the CNA_origin had a mean value of 0.8381, which was increased by 0.92 and 7.75% compared with LSTM and CNA_zhang, respectively. The results are shown in Figure 2.

The results showed that the sensitivity, accuracy, and specificity of UCEC were significantly lower than those of other tumors. The results of UCEC were further analyzed, and it was found that about 48–76% of UCEC samples were predicted to be OV, while 24–52% of UCEC samples were predicted to be BRCA. This may be because BRCA, OV, and UCEC are hormone-dependent tumors, which have a close relationship in tumorigenesis. Many reports have pointed out that BRCA, OV, and UCEC are related to changes in estrogen and estrogen receptors (Rodriguez et al., 2019; Scherbakov et al., 2019; Sehouli et al., 2019). Moreover, the physical location of ovary and uterus is very close, which may lead to contamination of tissue samples and difficulty in distinguishing UCEC from OV samples.

### 3.4. Impact of Sample Size

**Figure 3:**
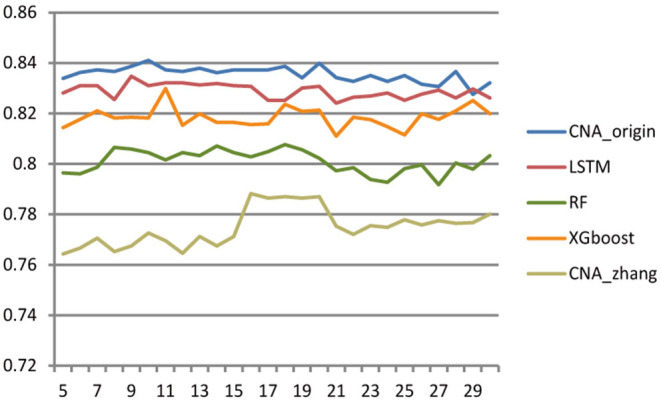
Effect of cross-validation fold k value on classifier performance. When the value of k became larger, the performance of classifiers was improved, but a small sample size of the test set had a negative impact on model evaluation.

Different cross-validation fold k values were used to study the effect of sample number on the performance of the classifier. The larger k was, the more samples there were in the training set, and then the fewer samples there were in the test set, and vice versa. The range of k ranged from 5 to 30 with step size = 1, and Figure 3 shows the accuracy of CNA_origin, LSTM, RF, XGboost, and CNA_origin with the different fold k values. With increasing k value, the performance of CNA_origin was gradually improved at first, which could be due to a bigger k including more training samples. But, as k became larger, the number of samples in the test set became smaller, and the performance of the classifiers was weakened. The results indicated that the performance of CNA_origin would be further improved if the training samples were expanded and that sufficient test samples were also very important for model evaluation.

### 3.5. Performance Comparison of Independent Datasets

**Figure 4:**
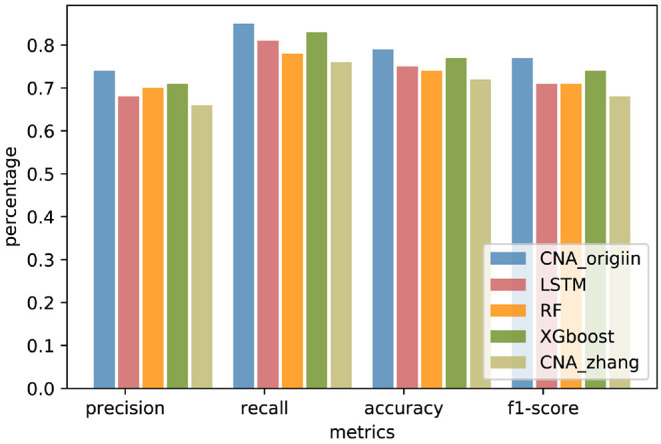
Performance comparison of CNA_origin and other algorithms (basic LSTM, RF, XGboost, and CNA_zhang) for independent datasets from the TCGA.

In order to compare generalization performance on the independent data, experiments were performed with CNA datasets released by TCGA in 2016 downloaded from http://gdac.broadinstitute.org/. The TCGA datasets had 1080 BRCA samples, 611 COADRAD samples, 577 GBM samples, 528 KIRC samples, 552 OV samples, and 533 UCEC samples, respectively. The preprocessing analysis of 24776 gene CNA values was performed with GISTIC2 (Mermel et al., 2011). The TCGA datasets were reasonably independent of the training data because of preprocessing analyses such as quality control, alignment, and variation detection, which had a different systematic bias. The genes involved in both MSKCC datasets and TCGA datasets were selected, and the TCGA samples existing in MSKCC datasets were removed. There were 19895 common genes present in the MSKCC and TCGA datasets, and the independent datasets contained 234 BRCA samples, 50 COADRAD samples, 25 GBM samples, 41 KIRC samples, 21 OV samples, and 99 UCEC samples (see Supplementary Material for details). The independent datasets were used to evaluate the performance of CNA_origin. As shown in Figure 4, the overall performance of CNA_origin in terms of precision, recall, accuracy, and F1-score was the highest among the tools, at 0.74, 0.85, 0.79, and 0.77, respectively (see Supplementary Material for details). According to the results shown in Figure 4, it was concluded that CNA_origin performed successfully in the independent datasets.

## 4. Conclusions

Patients with CUPS often have a low median survival time of 9–12 months. Precise diagnosis of the tissue origin for metastatic CUPS is essential for determining the treatment scheme to improve patient prognosis. A lot of studies have tried to use cancer biomarkers to predict the primary tumor site for CUPS so as to provide important guidelines for timely patient care and cancer therapy. CNA provides a new way to identify and classify tumor types. In this study, a computational method, CNA_origin, was proposed to predict the tissue of origin from information on gene CNA levels. CNA_origin set up an intellectual deep-learning network mainly composed of an autoencoder and a CNN. This predictor successfully learned the inherent information of gene copy number and exhibited superior performance to the classical algorithms on k-fold cross-validations and independent datasets.

At present, the accuracy of using only CNA as the biomarker for tumor traceability is not very high. Integrating multiple biomarkers, such as CNA and DNA methylation or gene expression data, to trace tumor is our future goal.

## Data Availability Statement

All datasets presented in this study are included in the article/ Supplementary Material.

## Author Contributions

YL conceived of the algorithm, develop the program, and wrote the manuscript. JY, BW, and GT helped with manuscript editing, designed, and performed experiments. PS and YW prepared the datasets. XL and CD carried out analyses and helped with the program design. HW designed of the work and participated in revising articles. All authors read and approved the final manuscript.

## Conflict of Interest

JY, CD, GT, and BW were employed by the company Geneis Beijing Co., Ltd. The remaining authors declare that the research was conducted in the absence of any commercial or financial relationships that could be construed as a potential conflict of interest.
