# Supplementary material for: A Deep Learning Framework to Predict Tumor Tissue-of-Origin Based on Copy Number Alteration
Source: Front Bioeng Biotechnol. 2020 Aug 5;8:701. doi: 10.3389/fbioe.2020.00701 (PMC7419421; doi:10.3389/fbioe.2020.00701)
Supplement: Supplementary file 1 [file Data_Sheet_1.PDF]

Supplementary Material

1 SUPPLEMENTARY DATA

The independent data were performed with CNA datasets released by TCGA in 2016, downloaded from http://gdac.broadinstitute.org/. The TCGA datasets had 1080 BRCA samples, 611 COADRAD samples, 577 GBM samples, 528 KIRC samples, 552 OV samples and 533 UCEC samples, respectively. After removing the samples existing in MSKCC datasets, there were 234 BRCA samples, 50 COADRAD samples, 25 GBM samples, 41 KIRC samples, 21 OV samples and 99 UCEC samples in the independent datasets.

BRCA:

TCGA-3C-AAAU-01A-11D-A41E-01, TCGA-3C-AALI-01A-11D-A41E-01, TCGA-3C-AALJ-01A-31D-A41E-01, TCGA-3C-AALK-01A-11D-A41E-01, TCGA-4H-AAAK-01A-12D-A41E-01, TCGA-5L-AAT0-01A-12D-A41E-01, TCGA-5L-AAT1-01A-12D-A41E-01, TCGA-5T-A9QA-01A-11D-A41E-01, TCGA-A2-A0CK-01A-11D-A227-01, TCGA-A2-A0CO-01A-13D-A227-01, TCGA-A2-A0CR-01A-11D-A227-01, TCGA-A2-A0EP-01A-52D-A22W-01, TCGA-A2-A3XS-01A-11D-A22W-01, TCGA-A2-A3XT-01A-11D-A22W-01, TCGA-A2-A3XU-01A-12D-A22W-01, TCGA-A2-A3XV-01A-21D-A238-01, TCGA-A2-A3XW-01A-11D-A238-01, TCGA-A2-A3XX-01A-21D-A238-01, TCGA-A2-A3XY-01A-11D-A238-01, TCGA-A2-A3XZ-01A-42D-A238-01, TCGA-A2-A3Y0-01A-11D-A238-01, TCGA-A2-A4RW-01A-21D-A25N-01, TCGA-A2-A4RX-01A-11D-A25N-01, TCGA-A2-A4RY-01A-31D-A25N-01, TCGA-A2-A4S0-01A-21D-A25N-01, TCGA-A2-A4S1-01A-21D-A25N-01, TCGA-A2-A4S2-01A-12D-A25N-01, TCGA-A2-A4S3-01A-21D-A25N-01, TCGA-A7-A0DC-01A-11D-A011-01, TCGA-A7-A13H-01A-11D-A227-01, TCGA-A7-A26E-01A-11D-A275-01, TCGA-A7-A26J-01A-11D-A275-01, TCGA-A7-A3RF-01A-11D-A227-01, TCGA-A7-A425-01A-11D-A242-01, TCGA-A7-A426-01A-22D-A242-01, TCGA-A7-A4SA-01A-11D-A25N-01, TCGA-A7-A4SB-01A-21D-A25N-01, TCGA-A7-A4SD-01A-11D-A25N-01, TCGA-A7-A4SE-01A-11D-A25N-01, TCGA-A7-A4SF-01A-11D-A25N-01, TCGA-A7-A56D-01A-11D-A27O-01, TCGA-A7-A5ZV-01A-11D-A28A-01, TCGA-A7-A5ZW-01A-12D-A29M-01, TCGA-A7-A5ZX-01A-12D-A29M-01, TCGA-A7-A6VV-01A-22D-A33D-01, TCGA-A7-A6VW-01A-21D-A33D-01, TCGA-A7-A6VX-01A-12D-A33D-01, TCGA-A7-A6VY-01A-12D-A33D-01, TCGA-AC-A3EH-01A-22D-A227-01, TCGA-AC-A3QP-01A-11D-A22W-01, TCGA-AC-A3QQ-01A-11D-A227-01, TCGA-AC-A3TM-01A-11D-A227-01, TCGA-AC-A3TN-01A-11D-A227-01, TCGA-AC-A3W5-01A-11D-A227-01, TCGA-AC-A3W6-01A-12D-A227-01, TCGA-AC-A3W7-01A-11D-A227-01, TCGA-AC-A3YI-01A-21D-A238-01, TCGA-AC-A3YJ-01A-11D-A22W-01, TCGA-AC-A4ZE-01A-11D-A41E-01, TCGA-AC-A5EH-01A-11D-A28A-01, TCGA-AC-A5EI-01A-11D-A27O-01, TCGA-AC-A5XS-01A-11D-A29M-01, TCGA-AC-A5XU-01A-11D-A28A-01, TCGA-AC-A62V-01A-11D-A31T-01, TCGA-AC-A62X-01A-11D-A29M-01, TCGA-AC-A62Y-01A-11D-A29M-01, TCGA-AC-A6IV-01A-12D-A33D-01, TCGA-AC-A6IW-01A-12D-A33D-01, TCGA-AC-A6IX-01A-12D-A32H-01, TCGA-AC-A6NO-01A-12D-A33D-01, TCGA-AC-A7VB-01A-11D-A350-01, TCGA-AC-A7VC-01A-11D-A350-01, TCGA-AC-A8OP-01A-11D-A36I-01, TCGA-AC-A8OQ-01A-11D-A41E-01, TCGA-AC-A8OR-01A-21D-A41E-01,

TCGA-AC-A8OS-01A-12D-A41E-01, TCGA-AQ-A54N-01A-11D-A25N-01, TCGA-AQ-A54O-01A-11D-A25N-01, TCGA-AQ-A7U7-01A-22D-A350-01, TCGA-AR-A1AM-01A-41D-A227-01, TCGA-AR-A5QM-01A-11D-A27O-01, TCGA-AR-A5QN-01A-12D-A28A-01, TCGA-AR-A5QP-01A-11D-A28A-01, TCGA-AR-A5QQ-01A-11D-A28A-01, TCGA-B6-A3ZX-01A-11D-A238-01, TCGA-B6-A400-01A-11D-A238-01, TCGA-B6-A401-01A-11D-A238-01, TCGA-B6-A402-01A-11D-A238-01, TCGA-B6-A408-01A-12D-A242-01, TCGA-B6-A409-01A-11D-A242-01, TCGA-B6-A40B-01A-11D-A238-01, TCGA-B6-A40C-01A-11D-A238-01, TCGA-BH-A0B2-01A-11D-A10L-01, TCGA-BH-A0HY-01A-11D-A059-01, TCGA-BH-A28O-01A-11D-A227-01, TCGA-BH-A42T-01A-11D-A242-01, TCGA-BH-A42U-01A-12D-A242-01, TCGA-BH-A42V-01A-11D-A242-01, TCGA-BH-A5IZ-01A-11D-A27O-01, TCGA-BH-A5J0-01A-11D-A27O-01, TCGA-BH-A6R8-01A-21D-A33D-01, TCGA-BH-A6R9-01A-21D-A32H-01, TCGA-BH-A8FY-01A-11D-A36I-01, TCGA-BH-A8FZ-01A-11D-A350-01, TCGA-BH-A8G0-01A-11D-A350-01, TCGA-BH-AB28-01A-31D-A41E-01, TCGA-C8-A12O-01A-11D-A111-01, TCGA-C8-A8HP-01A-11D-A36I-01, TCGA-C8-A8HQ-01A-11D-A36I-01, TCGA-C8-A8HR-01A-11D-A36I-01, TCGA-C8-A9FZ-01A-11D-A41E-01, TCGA-D8-A140-01A-11D-A111-01, TCGA-D8-A3Z5-01A-41D-A242-01, TCGA-D8-A3Z6-01A-11D-A238-01, TCGA-D8-A4Z1-01A-21D-A25N-01, TCGA-D8-A73U-01A-11D-A33D-01, TCGA-D8-A73W-01A-22D-A350-01, TCGA-D8-A73X-01A-11D-A32H-01, TCGA-E2-A14T-01A-11D-A111-01, TCGA-E2-A14U-01A-11D-A227-01, TCGA-E2-A15D-01A-11D-A111-01, TCGA-E2-A15F-01A-11D-A111-01, TCGA-E2-A15S-01A-11D-A111-01, TCGA-E2-A15T-01A-11D-A111-01, TCGA-E2-A56Z-01A-12D-A29M-01, TCGA-E2-A570-01A-11D-A29M-01, TCGA-E2-A573-01A-11D-A29M-01, TCGA-E2-A574-01A-11D-A29M-01, TCGA-E2-A576-01A-11D-A31T-01, TCGA-E2-A9RU-01A-11D-A41E-01, TCGA-E9-A3QA-01A-61D-A227-01, TCGA-E9-A3X8-01A-31D-A22W-01, TCGA-E9-A54X-01A-11D-A25N-01, TCGA-E9-A54Y-01A-11D-A25N-01, TCGA-E9-A5FK-01A-11D-A27O-01, TCGA-E9-A5FL-01A-11D-A27O-01, TCGA-E9-A5UO-01A-11D-A28A-01, TCGA-E9-A5UP-01A-11D-A28A-01, TCGA-E9-A6HE-01A-11D-A31T-01, TCGA-EW-A3U0-01A-11D-A227-01, TCGA-EW-A423-01A-11D-A242-01, TCGA-EW-A424-01A-11D-A242-01, TCGA-EW-A6S9-01A-22D-A33D-01, TCGA-EW-A6SA-01A-21D-A32H-01, TCGA-EW-A6SB-01A-12D-A32H-01, TCGA-EW-A6SC-01A-12D-A32H-01, TCGA-EW-A6SD-01A-12D-A33D-01, TCGA-GM-A3NW-01A-21D-A227-01, TCGA-GM-A3XG-01A-31D-A242-01, TCGA-GM-A3XL-01A-11D-A22W-01, TCGA-GM-A3XN-01A-12D-A22W-01, TCGA-GM-A4E0-01A-12D-A25N-01, TCGA-GM-A5PV-01A-11D-A28A-01, TCGA-GM-A5PX-01A-12D-A28A-01, TCGA-HN-A2OB-01A-21D-A27O-01, TCGA-JL-A3YW-01A-12D-A238-01, TCGA-JL-A3YX-01A-11D-A22W-01, TCGA-LD-A66U-01A-11D-A31T-01, TCGA-LD-A74U-01A-13D-A33D-01, TCGA-LD-A7W5-01A-22D-A350-01, TCGA-LD-A7W6-01A-81D-A350-01, TCGA-LD-A9QF-01A-32D-A41E-01, TCGA-LL-A440-01A-11D-A242-01, TCGA-LL-A441-01A-11D-A242-01, TCGA-LL-A442-01A-11D-A242-01, TCGA-LL-A50Y-01A-11D-A25N-01, TCGA-LL-A5YL-01A-12D-A29M-01, TCGA-LL-A5YM-01A-11D-A28A-01, TCGA-LL-A5YN-01A-11D-A28A-01, TCGA-LL-A5YO-01A-21D-A28A-01, TCGA-LL-A5YP-01A-21D-A28A-01, TCGA-LL-A6FP-01A-11D-A31T-01, TCGA-LL-A6FQ-01A-11D-A31T-01, TCGA-LL-A6FR-01A-12D-A31T-01, TCGA-LL-A73Y-01A-11D-A33D-01, TCGA-LL-A73Z-01A-11D-A32H-01, TCGA-LL-A740-01A-21D-A32H-01, TCGA-LL-A7SZ-01A-32D-A350-01, TCGA-LL-A7T0-01A-31D-A350-01, TCGA-LL-A8F5-01A-11D-A36I-01, TCGA-LL-A9Q3-01A-11D-A41E-01, TCGA-LQ-A4E4-01A-11D-A25N-01, TCGA-MS-A51U-01A-31D-A25N-01, TCGA-OK-A5Q2-01A-11D-A27O-01, TCGA-OL-A5D6-01A-21D-A27O-01, TCGA-OL-A5D7-01A-11D-A27O-01, TCGA-OL-A5D8-01A-11D-A27O-01, TCGA-OL-A5DA-01A-11D-A27O-01, TCGA-OL-A5RU-01A-11D-A28A-01, TCGA-OL-A5RV-01A-12D-A28A-01,

TCGA-OL-A5RW-01A-11D-A28A-01, TCGA-OL-A5RX-01A-11D-A28A-01, TCGA-OL-A5RY-01A-21D-A28A-01, TCGA-OL-A5RZ-01A-11D-A28A-01, TCGA-OL-A5S0-01A-11D-A28A-01, TCGA-OL-A66H-01A-11D-A29M-01, TCGA-OL-A66I-01A-21D-A29M-01, TCGA-OL-A66J-01A-11D-A29M-01, TCGA-OL-A66K-01A-11D-A29M-01, TCGA-OL-A66L-01A-12D-A31T-01, TCGA-OL-A66N-01A-12D-A31T-01, TCGA-OL-A66O-01A-11D-A31T-01, TCGA-OL-A66P-01A-11D-A31T-01, TCGA-OL-A6VO-01A-12D-A33D-01, TCGA-OL-A6VQ-01A-12D-A41E-01, TCGA-OL-A6VR-01A-32D-A33D-01, TCGA-OL-A97C-01A-32D-A41E-01, TCGA-PE-A5DC-01A-12D-A27O-01, TCGA-PE-A5DD-01A-12D-A27O-01, TCGA-PE-A5DE-01A-11D-A27O-01, TCGA-PL-A8LV-01A-21D-A41E-01, TCGA-PL-A8LX-01A-11D-A41E-01, TCGA-PL-A8LY-01A-11D-A41E-01, TCGA-PL-A8LZ-01A-31D-A36I-01, TCGA-S3-A6ZF-01A-32D-A32H-01, TCGA-S3-A6ZG-01A-22D-A32H-01, TCGA-S3-A6ZH-01A-22D-A32H-01, TCGA-S3-AA0Z-01A-11D-A41E-01, TCGA-S3-AA10-01A-21D-A41E-01, TCGA-S3-AA11-01A-31D-A41E-01, TCGA-S3-AA12-01A-11D-A41E-01, TCGA-S3-AA14-01A-11D-A41E-01, TCGA-S3-AA15-01A-11D-A41E-01, TCGA-S3-AA17-01A-11D-A41E-01, TCGA-UL-AAZ6-01A-11D-A41E-01, TCGA-UU-A93S-01A-21D-A41E-01, TCGA-V7-A7HQ-01A-11D-A33D-01, TCGA-W8-A86G-01A-21D-A36I-01, TCGA-WT-AB41-01A-11D-A41E-01, TCGA-WT-AB44-01A-11D-A41E-01, TCGA-XX-A899-01A-11D-A36I-01, TCGA-XX-A89A-01A-11D-A36I-01, TCGA-Z7-A8R5-01A-42D-A41E-01, TCGA-Z7-A8R6-01A-11D-A41E-01

COADREAD:

TCGA-3L-AA1B-01A-11D-A36W-01, TCGA-4N-A93T-01A-11D-A36W-01, TCGA-4T-AA8H-01A-11D-A40O-01, TCGA-5M-AAT4-01A-11D-A40O-01, TCGA-5M-AAT5-01A-21D-A40O-01, TCGA-5M-AAT6-01A-11D-A40O-01, TCGA-5M-AATA-01A-31D-A40O-01, TCGA-5M-AATE-01A-11D-A40O-01, TCGA-A6-2677-01A-01D-A274-01, TCGA-A6-2684-01A-01D-A274-01, TCGA-A6-3809-01A-01D-A274-01, TCGA-A6-3810-01A-01D-A274-01, TCGA-A6-5656-01A-21D-A274-01, TCGA-A6-5659-01A-01D-A274-01, TCGA-A6-6650-01A-11D-A274-01, TCGA-A6-6780-01A-11D-A274-01, TCGA-A6-6781-01A-22D-A274-01, TCGA-A6-A565-01A-31D-A28F-01, TCGA-A6-A566-01A-11D-A28F-01, TCGA-A6-A567-01A-31D-A28F-01, TCGA-A6-A56B-01A-31D-A28F-01, TCGA-A6-A5ZU-01A-11D-A28F-01, TCGA-AD-A5EJ-01A-11D-A28F-01, TCGA-AD-A5EK-01A-11D-A28F-01, TCGA-AF-A56K-01A-32D-A38W-01, TCGA-AF-A56L-01A-31D-A38W-01, TCGA-AF-A56N-01A-12D-A38W-01, TCGA-AY-A54L-01A-11D-A28F-01, TCGA-AY-A69D-01A-11D-A36W-01, TCGA-AY-A71X-01A-12D-A36W-01, TCGA-AY-A8YK-01A-11D-A40O-01, TCGA-NH-A50T-01A-11D-A28F-01, TCGA-NH-A50U-01A-33D-A36W-01, TCGA-NH-A50V-01A-11D-A28F-01, TCGA-NH-A5IV-01A-42D-A36W-01, TCGA-NH-A6GA-01A-11D-A36W-01, TCGA-NH-A6GB-01A-11D-A36W-01, TCGA-NH-A6GC-01A-12D-A40O-01, TCGA-NH-A8F7-01A-11D-A40O-01, TCGA-NH-A8F8-01A-72D-A40O-01, TCGA-QG-A5YV-01A-11D-A28F-01, TCGA-QG-A5YW-01A-11D-A28F-01, TCGA-QG-A5YX-01A-11D-A28F-01, TCGA-QG-A5Z1-01A-11D-A28F-01, TCGA-QG-A5Z2-01A-11D-A28F-01, TCGA-QL-A97D-01A-12D-A40O-01, TCGA-RU-A8FL-01A-11D-A36W-01, TCGA-SS-A7HO-01A-21D-A36W-01, TCGA-T9-A92H-01A-11D-A36W-01, TCGA-WS-AB45-01A-11D-A40O-01

KIRC:

TCGA-3Z-A93Z-01A-11D-A36W-01, TCGA-6D-AA2E-01A-11D-A36W-01, TCGA-A3-A6NI-01A-11D-A33B-01, TCGA-A3-A6NJ-01A-12D-A33B-01, TCGA-A3-A6NL-01A-11D-A33B-01, TCGA-A3-A6NN-01A-12D-A33B-01, TCGA-A3-A8CQ-01A-11D-A36W-01, TCGA-A3-A8OU-01A-11D-A36W-01, TCGA-A3-A8OV-01A-11D-A36W-01, TCGA-A3-A8OW-01A-11D-A36W-01, TCGA-A3-A8OX-01A-11D-A36W-01, TCGA-B2-3924-01A-02D-A274-01, TCGA-B2-5633-01A-01D-A274-01,

TCGA-B2-5635-01A-01D-A274-01, TCGA-B2-A4SR-01A-11D-A25U-01, TCGA-B8-A54D-01A-21D-A25U-01, TCGA-B8-A54E-01A-11D-A25U-01, TCGA-B8-A54F-01A-11D-A25U-01, TCGA-B8-A54G-01A-11D-A25U-01, TCGA-B8-A54H-01A-11D-A33B-01, TCGA-B8-A54I-01A-21D-A33B-01, TCGA-B8-A54J-01A-11D-A33B-01, TCGA-B8-A54K-01A-11D-A33B-01, TCGA-B8-A7U6-01A-12D-A36W-01, TCGA-B8-A8YJ-01A-13D-A38W-01, TCGA-BP-4338-01A-01D-1283-01, TCGA-BP-4762-01A-02D-1283-01, TCGA-BP-4769-01A-01D-1283-01, TCGA-DV-A4VX-01A-11D-A25U-01, TCGA-DV-A4VZ-01A-11D-A25U-01, TCGA-DV-A4W0-01A-11D-A25U-01, TCGA-G6-A5PC-01A-11D-A33B-01, TCGA-G6-A8L6-01A-11D-A36W-01, TCGA-G6-A8L7-01A-11D-A36W-01, TCGA-G6-A8L8-01A-21D-A36W-01, TCGA-GK-A6C7-01A-11D-A33B-01, TCGA-MM-A563-01A-11D-A25U-01, TCGA-MM-A564-01A-11D-A25U-01, TCGA-MM-A84U-01A-11D-A36W-01, TCGA-MW-A4EC-01A-11D-A25U-01, TCGA-T7-A92I-01A-11D-A36W-01 ,

GBM:

TCGA-06-0122-01A-01D-0214-01, TCGA-06-0124-01A-01D-0214-01, TCGA-06-0125-01A-01D-0214-01, TCGA-06-0126-01A-01D-0214-01, TCGA-06-0128-01A-01D-0214-01, TCGA-06-0130-01A-01D-0214-01, TCGA-06-0141-01A-01D-0214-01, TCGA-06-0143-01A-01D-0214-01, TCGA-06-0166-01A-01D-0236-01, TCGA-06-A5U0-01A-11D-A33S-01, TCGA-06-A5U1-01A-11D-A33S-01, TCGA-06-A6S0-01A-11D-A33S-01, TCGA-06-A6S1-01A-11D-A33S-01, TCGA-06-A7TK-01A-21D-A390-01, TCGA-06-A7TL-01A-11D-A390-01, TCGA-19-A60I-01A-12D-A33S-01, TCGA-19-A6J4-01A-11D-A33S-01, TCGA-19-A6J5-01A-21D-A33S-01, TCGA-4W-AA9R-01A-11D-A390-01, TCGA-4W-AA9S-01A-11D-A390-01, TCGA-4W-AA9T-01A-11D-A390-01, TCGA-OX-A56R-01A-11D-A33S-01, TCGA-RR-A6KA-01A-21D-A33S-01, TCGA-RR-A6KB-01A-12D-A33S-01, TCGA-RR-A6KC-01A-31D-A33S-01

OV:

TCGA-13-1819-01A-01D-2069-01, TCGA-13-A5FT-01A-11D-A402-01, TCGA-13-A5FU-01A-11D-A402-01, TCGA-29-A5NZ-01A-11D-A402-01, TCGA-3P-A9WA-01A-11D-A402-01, TCGA-59-A5PD-01A-11D-A402-01, TCGA-5X-AA5U-01A-11D-A402-01, TCGA-72-4231-01A-01D-1017-01, TCGA-72-4232-01A-01D-1017-01, TCGA-72-4233-01A-01D-1017-01, TCGA-72-4234-01A-01D-1017-01, TCGA-72-4235-01A-01D-1017-01, TCGA-72-4236-01A-01D-1017-01, TCGA-72-4237-01A-01D-1017-01, TCGA-72-4238-01A-01D-1017-01, TCGA-72-4240-01A-01D-1017-01, TCGA-72-4241-01A-01D-1017-01, TCGA-OY-A56P-01A-12D-A402-01, TCGA-OY-A56Q-01A-11D-A402-01, TCGA-VG-A8LO-01A-11D-A402-01, TCGA-WR-A838-01A-12D-A402-01

UCEC:

TCGA-2E-A9G8-01A-11D-A402-01, TCGA-4E-A92E-01A-11D-A37M-01, TCGA-5B-A90C-01A-11D-A37M-01, TCGA-5S-A9Q8-01A-11D-A402-01, TCGA-A5-A1OH-01A-21D-A227-01, TCGA-A5-A3LO-01A-11D-A227-01, TCGA-A5-A3LP-01A-11D-A227-01, TCGA-A5-A7WJ-01A-12D-A34P-01, TCGA-A5-A7WK-01A-11D-A34P-01, TCGA-A5-AB3J-01A-11D-A402-01, TCGA-AJ-A23N-01A-11D-A227-01, TCGA-AJ-A3I9-01A-11D-A227-01, TCGA-AJ-A3NC-01A-11D-A227-01, TCGA-AJ-A3NE-01A-11D-A227-01, TCGA-AJ-A3NF-01A-11D-A227-01, TCGA-AJ-A3NG-01A-11D-A227-01, TCGA-AJ-A3NH-01A-11D-A227-01, TCGA-AJ-A3OJ-01A-11D-A227-01, TCGA-AJ-A3OK-01A-12D-A227-01, TCGA-AJ-A3OL-01A-11D-A227-01, TCGA-AJ-A3QS-01A-11D-A227-01, TCGA-AJ-A3TW-01A-11D-A227-01, TCGA-AJ-A5DV-01A-11D-A27O-01, TCGA-AJ-A5DW-01A-11D-A27O-01, TCGA-AJ-A6NU-01A-11D-A34P-01, TCGA-AJ-A8CT-01A-11D-A37M-01, TCGA-AJ-A8CV-01A-11D-A37M-01,

TCGA-AJ-A8CW-01A-11D-A37M-01, TCGA-AP-A5FX-01A-11D-A27O-01, TCGA-AX-A3FS-01A-11D-A227-01, TCGA-AX-A3FT-01A-11D-A227-01, TCGA-AX-A3FV-01A-11D-A227-01, TCGA-AX-A3FW-01A-11D-A227-01, TCGA-AX-A3FX-01A-11D-A227-01, TCGA-AX-A3FZ-01A-11D-A227-01, TCGA-AX-A3G1-01A-11D-A227-01, TCGA-AX-A3G8-01A-11D-A227-01, TCGA-AX-A3G9-01A-11D-A227-01, TCGA-AX-A3GB-01A-11D-A227-01, TCGA-B5-A3F9-01A-21D-A227-01, TCGA-B5-A3FC-01A-11D-A227-01, TCGA-B5-A3S1-01A-11D-A227-01, TCGA-B5-A5OC-01A-21D-A27O-01, TCGA-B5-A5OD-01A-11D-A31T-01, TCGA-B5-A5OE-01A-11D-A31T-01, TCGA-BG-A3EW-01A-11D-A227-01, TCGA-BG-A3PP-01A-11D-A227-01, TCGA-BK-A0CA-01A-21D-A275-01, TCGA-BK-A0CC-01A-21D-A275-01, TCGA-BK-A139-01A-11D-A275-01, TCGA-BK-A13B-01A-51D-A227-01, TCGA-BK-A26L-01A-11D-A275-01, TCGA-BK-A4ZD-01A-11D-A27O-01, TCGA-BK-A56F-01A-32D-A27O-01, TCGA-BK-A6W3-01A-12D-A34P-01, TCGA-BK-A6W4-01A-12D-A34P-01, TCGA-D1-A3JP-01A-31D-A227-01, TCGA-D1-A3JQ-01A-11D-A227-01, TCGA-E6-A8L9-01A-21D-A37M-01, TCGA-EO-A3KU-01A-11D-A227-01, TCGA-EO-A3KW-01A-11D-A227-01, TCGA-EO-A3KX-01A-11D-A227-01, TCGA-EO-A3L0-01A-11D-A227-01, TCGA-EY-A3L3-01A-11D-A227-01, TCGA-EY-A3QX-01A-11D-A227-01, TCGA-EY-A4KR-01A-11D-A27O-01, TCGA-EY-A547-01A-11D-A27O-01, TCGA-EY-A548-01A-11D-A27O-01, TCGA-EY-A549-01A-11D-A27O-01, TCGA-EY-A54A-01A-11D-A27O-01, TCGA-EY-A5W2-01A-11D-A31T-01, TCGA-EY-A72D-01A-12D-A34P-01, TCGA-FI-A3PV-01A-11D-A227-01, TCGA-FI-A3PX-01A-11D-A227-01, TCGA-JU-AAVI-01A-11D-A402-01, TCGA-K6-A3WQ-01A-11D-A227-01, TCGA-KJ-A3U4-01A-11D-A227-01, TCGA-KP-A3VZ-01A-11D-A227-01, TCGA-KP-A3W0-01A-21D-A227-01, TCGA-KP-A3W1-01A-11D-A227-01, TCGA-KP-A3W3-01A-11D-A227-01, TCGA-KP-A3W4-01A-11D-A227-01, TCGA-PG-A5BC-01A-12D-A27O-01, TCGA-PG-A6IB-01A-21D-A31T-01, TCGA-PG-A7D5-01A-11D-A34P-01, TCGA-PG-A914-01A-11D-A37M-01, TCGA-PG-A915-01A-11D-A37M-01, TCGA-PG-A916-01A-11D-A37M-01, TCGA-PG-A917-01A-31D-A37M-01, TCGA-QF-A5YS-01A-11D-A31T-01, TCGA-QF-A5YT-01A-11D-A31T-01, TCGA-QS-A5YQ-01A-11D-A31T-01, TCGA-QS-A5YR-01A-31D-A31T-01, TCGA-QS-A744-01A-11D-A34P-01, TCGA-QS-A8F1-01A-21D-A37M-01, TCGA-SJ-A6ZI-01A-12D-A34P-01, TCGA-SJ-A6ZJ-01A-12D-A34P-01, TCGA-SL-A6J9-01A-11D-A31T-01, TCGA-SL-A6JA-01A-11D-A31T-01

2 SUPPLEMENTARY TABLES AND FIGURES

The overall performance of CNA origin in terms of precision, recall, accuracy and F1-score were sumarized in Table S1

Table S1. The performance comparison of CNA origin and other algorithms (basic LSTM, RF, XGboost and CNA_zhang) for independent datasets from the TCGA

predictor	Precision	Recall	F1-score	Accuracy
CNA_origin	0.7367	0.8486	0.7652	0.7468
LSTM	0.6797	0.8096	0.7116	0.7468
RF	0.6964	0.7775	0.7056	0.7404
XGboost	0.7106	0.8263	0.7412	0.7723
CNA_zhang	0.6604	0.7572	0.6794	0.7191

According to the results as shown in Table S1 , it was concluded that CNA origin successfully performed in the independent datasets.
